# Cost of Sauti ya Vijana (SYV), a mental health intervention for young people living with HIV in Tanzania: Results from a pilot randomized controlled trial

**DOI:** 10.1371/journal.pgph.0005397

**Published:** 2025-12-18

**Authors:** Ayodamope Fawole, Armand Zimmerman, Moses Bateganya, Blandina T. Mmbaga, Dorothy Dow, Osondu Ogbuoji

**Affiliations:** 1 Duke Center for Policy Impact in Global Health, Duke Global Health Institute, Duke University, Durham, North Carolina, United States of America; 2 Duke Global Health Institute, Duke University, Durham, North Carolina, United States of America; 3 United States Agency for International Development (USAID), Dar Es Salaam, Tanzania; 4 Kilimanjaro Christian Medical University College, Moshi, Tanzania; 5 Kilimanjaro Christian Medical Center-Duke Collaboration, Moshi, Tanzania; 6 Kilimanjaro Clinical Research Institute, Moshi, Tanzania; 7 Department of Pediatrics, Duke University Medical Center, Durham, North Carolina, United States of America; 8 Department of Population Health Sciences, Duke University, Durham, North Carolina, United States of America; 9 Margolis Institute for Healthcare Policy, Duke University, Durham, North Carolina, United States of America; University of Embu, KENYA

## Abstract

Sauti ya Vijana (SYV) is a ten-session, group-based mental health and life skills intervention targeting young people living with HIV (YPLWH) in Tanzania. By addressing mental health distress and promoting self-efficacy and resilience to HIV-related stigma and disclosure, SYV aims to improve adherence to antiretroviral therapy (ART) and virologic suppression. A pilot randomized controlled trial (RCT) of SYV was conducted in Moshi, Tanzania, from April 2016 to August 2020. We aimed to estimate the cost of SYV delivery in the pilot RCT, identify key cost drivers, and inform future scale-up. We used a hybrid costing methodology and an intent-to-treat approach to estimate the cost of SYV delivery in the two-arm pilot RCT. Cost data were obtained from project records and interviews with key project personnel. Our estimates included start-up, service delivery, research-related costs, and program administrative fees. Human resources were costed in terms of full-time equivalents for salaried personnel. Costs are reported in 2022 USD. The pilot study included 58 participants in the SYV arm and 47 in the standard-of-care arm. The total cost to deliver SYV to the 58 participants in the SYV arm was approximately US$137,618·05. The total per-participant cost was US$2,372·72, the total non research cost was US$56,111·70, and the non-research cost per participant was US$967·44. Research-related costs comprised 59·23% (US$81,506·35) of the total cost. The most significant individual drivers of the total cost were research-related ART concentration in hair tests used to measure adherence (US$42,136·43), salaries for group leaders (US$33,607·94), and viral load tests (US$22,385·55). Our findings show that research-related expenses made up over half of the total costs of SYV delivery. Thus, a scale-up of the intervention without the additional trial components for measuring intervention efficacy would have better budgetary implications due to a smaller research footprint. These findings should guide policymakers in expanding SYV and similar mental health interventions for YPLWH.

## Introduction

Despite the advancements in antiretroviral therapy (ART), HIV continues to pose a significant health and economic burden to society [1]. In particular, young people living with HIV (YPLWH) are at high risk and constitute a growing proportion of people living with HIV [2]. Globally, five million young people live with HIV and over 2,400 young people become newly infected daily [3]. YPLWH have to navigate challenges such as societal stigma and peer relationships, which contribute to reduced adherence to ART and poorer health outcomes. Previous studies have found that YPLWH have lower rates of ART adherence, retention in care, and virologic suppression compared to older adults [4–6].

Moreover, research indicates that up to half of YPLWH may experience mental health challenges including elevated rates of depression, anxiety, and suicidal ideation. These challenges are further compounded by the psychosocial impact of AIDS on their families, such as caring for critically ill relatives, losing family members to AIDS, and enduring poverty [7]. Together, these findings underscore the need for tailored mental health interventions that address both the psychosocial and adherence challenges faced by YPLWH.

Tanzania has a large and growing number of YPLWH and currently accounts for approximately six percent of the global population of adolescents living with HIV [8]. In 2022, 10,000 new HIV infections and 2,300 HIV related deaths are estimated to have occurred in the 15–24 age group [9]. Given that over 50% of the Tanzanian population is under the age of 18 years and 70% under 30 years, these statistics underline the public health crisis, further emphasizing the need for targeted health interventions and policies to mitigate the impact of HIV on this demographic [10].

Sauti ya Vijana (SYV), a mental health and life skills intervention which translates to ‘Voice of the Youth,’ was developed to address mental health challenges and improve the health outcomes among YPLWH. Addressing mental health and psychosocial challenges related to stigma and disclosure is crucial to improving health outcomes in YPLWH. The intervention is delivered by trained young adult group leaders who live with HIV and understand the needs of YPLWH [11].

Previous interventions, such as MEMA kwa Vijana, a behavioral intervention aimed at reducing the risk of HIV acquisition among young people in Tanzania yielded positive results but did not specifically address the challenges faced by those already living with HIV [12]. SYV, explicitly targets YPLWH and incorporates a dedicated mental health curriculum. Additionally, targeted psychosocial programs, such as an enhanced peer supporter intervention for young mothers living with HIV, have been developed; however, to our knowledge, they have not been evaluated through a controlled trial [13]. Results from the pilot randomized controlled trial (RCT) of SYV, conducted between April 2016 and August 2020, suggest that it not only promotes self-efficacy and resilience to HIV-related stigma and disclosure in YPLWH but also could improve participants’ adherence to ART and virologic suppression [14]. Based on these promising results, the SYV intervention is being scaled up in Tanzania, highlighting the need for reliable cost estimates.

While previous studies have examined the costs of various HIV interventions, few have examined the cost of mental health interventions for YPLWH in low- and middle-income countries [14,15,16]. In this study, we conducted a retrospective cost analysis of the SYV pilot trial and estimated the cost of delivering SYV to its beneficiaries. The results inform the feasibility of a scale-up, provides a cost benchmark for implementing mental health interventions targeting YPLWH in sub-Saharan Africa, lays the foundation for future economic evaluations, and offers policy and programmatic planning guidance.

## Methods

### Study population, settings, and location

The SYV study population consisted of YPLWH aged 12–24 (mean age of 18 years) who were aware of their HIV status. Notably, approximately 86% of the participants were reported to have been perinatally infected with HIV. Participants were recruited from two adolescent HIV clinics, the Kilimanjaro Christian Medical Centre (KCMC) and the Mawenzi Regional Referral Hospital in the Kilimanjaro region, Moshi, Tanzania. The intervention was delivered to all participants at Kilimanjaro Clinical Research Institute branch in the Majengo neighborhood of Moshi, Tanzania, a facility centrally located and away from the clinics where they received ART [17].

### Pilot RCT design

The SYV pilot RCT was an individually randomized group treatment trial conducted from April 2016 to August 2020 [18]. The trial used a stepped-wedge design with three randomized waves and three crossover waves. Eligibility criteria included young people ages 12–24 years living with HIV and receiving ART. Participants were randomized to receive either the standard of care (SOC) or the SYV intervention in addition to SOC. Participants randomized to the SOC arm had the option of receiving the SYV intervention in one of three crossover waves after the intervention arm was complete. The intervention was delivered to the first SOC group following the third wave of the SYV intervention group and then sequentially to two other SOC groups [19].

**Fig 1:**
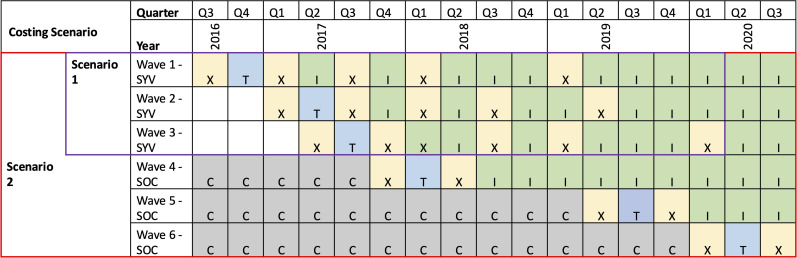
SYV stepped-wedge study design and costing approach. (Legend: X or Yellow = quantitative data collection from study participants, T or Blue = Ten-week delivery of SYV intervention, I or Green = Indicates study wave has received the SYV intervention, C or Grey = Indicates RCT control arm).

Over approximately ten weeks, participants in the SYV arm received ten 90-minute group-based sessions and two individual-based sessions, which used evidence-based components of cognitive behavioral therapy, interpersonal psychotherapy, and motivational interviewing to improve mental health, treatment adherence, and virologic suppression among participants. All SYV sessions were delivered by young adult group leaders ages 23–30 years who were living with or affected by HIV, some with prior experience delivering a mental health intervention for orphaned children. Six SYV group leaders completed an intensive two-week training delivered by the principal investigator and a US-based clinical psychologist. Each intervention session was led by two group leaders, while a third monitored fidelity using a checklist and took session notes. Weekly supervision meetings were held in person with the principal investigator and remotely via Skype with the US-based clinical psychologist [18]. Participants in the SOC arm received routine adherence counseling and HIV care per Tanzania’s national guidelines for the management of HIV [20]. Data were collected at baseline, 6, 12, 18, and 30 months for participants in both arms and pre- and post-intervention for the SOC crossover waves (Fig 1). Data collection included quantitative measures of demographics, reported risk behaviors, mental health, stigma, ART adherence, and viral load results.

### Costing perspective, time horizon, and discount rates

We conducted the cost analysis from a healthcare provider perspective, estimating only the incremental costs of the SYV program, including key intervention expenses such as group leader salaries, training costs, facilities, and supervisor salaries. Costs associated with the HIV/AIDS clinical care, the health facility or other existing infrastructure on which the program was built were excluded from this analysis. Both financial costs and real-world costs were incorporated into our analysis. All expenses are presented in 2022 US dollars, with program costs reported at 0% discount rate. We provide estimates of the total annual program costs discounted at an annual rate of 3% in the appendix (see Tables A and B in S1 Appendix). Our study had a time horizon of four years and five months or 53 months, corresponding to the period the RCT was conducted – April 2016 to August 2020.

### Costing scenarios

We report the total and per-participant costs of delivering the SYV intervention in the pilot RCT, including both intervention start-up and implementation. We provide cost estimates for two scenarios (Fig 1). In the first scenario, we calculate the costs for SYV intervention to 58 participants who were initially randomized to the intervention arm (the first three waves of intervention delivery). In the second scenario, we calculate the costs of SYV intervention delivery and follow-up to all 105 participants, including 47 SOC participants who received the intervention in three additional crossover waves.

Data collection for SYV participants occurred at five time points (baseline, 6, 12, 18, and 30 months), while for SOC crossover wave participants, data were collected afterwards at two time points (baseline and post-intervention). However, cost estimates are calculated for the entire intervention period in each scenario, not at each data collection time point. Consequently, 58 participants in the SYV arm had 290 study visits, and 47 participants in the SOC arm had 94 study visits, leading to a total of 384 study visits across both arms. Both scenarios have a similar time horizon of approximately four and a half years due to the staggered start and the longer follow-up period in the first three SYV intervention group waves. Our analysis uses an intent-to-treat approach whereby we assume (1) all participants randomized to the SYV or SOC arm received their assigned treatment, (2) all participants in the SOC arm received the SYV intervention in the crossover waves, and (3) data were collected at five time points for the SYV participants and two time points for the SOC participants.

We included both research and non research costs in calculating total program costs. However, only the breakdown of non research trial activities is presented in the manuscript (Table 1). Unit costs and resource utilization data for items and activities related SYV delivery were collected from RCT financial records and interviews with RCT key personnel. Tables 2 and 3 show the cost and resource use for all non research trial activities (see Tables C to E in S1 Appendix for all research related trial costs). To calculate the total cost of each item/activity included in the analysis, we multiplied the unit costs by the quantity consumed. Per participant costs were calculated by dividing the total cost of each item/activity by the total number of participants who received the SYV intervention for the given scenario.

**Table 1:** Non research costing parameters.

Item	Item definition	Cost/unit	Data source
**Personnel**			
Trainer salary	Annual salary paid to individuals who trained the group leaders	$7,060·86/FTE	RCT Key personnel interviews
Group leader salary	Annual salary paid to group leaders who delivered the intervention	$3,981·28/FTE	RCT financial records
Site supervisor salary	Annual salary paid to supervisor overseeing research site	$4,272·12/FTE	RCT Key personnel interviews
**Facilities**			
Building rent for training space	Rent for facility space used to conduct group leader trainings	$81·88/week	RCT Key personnel interviews
Building rent for intervention delivery space	Rent for facility space used to deliver the intervention	$237·34/month	RCT financial records
**Utilities**			
Internet	Internet service	$1·63/week	RCT Key personnel interviews
Phone calls	Follow-up phone calls to participants	$3·59/participant	RCT financial records and key personnel interviews
**Printing/office**			
Writing utensils	Writing materials used to deliver the intervention	$9·62/month	RCT financial records
SYV manuals	Manuals to facilitate group leader trainings	$94·94/group leader	RCT Key personnel interviews
Handouts	Participant handouts	$2·37/participant	RCT Key personnel interviews
**Compensation**			
Participant food vouchers	Food vouchers given to participants per SYV intervention wave	$14·47/participant	RCT financial records
Participant travel vouchers	Travel vouchers given to participants per SYV intervention wave	$30·80/participant	RCT financial records
Trainer travel allowance	Allowance provided for a U.S.-based trainer traveling to conduct training for group leaders	$1,780·00/trainer	RCT financial records
Training snacks and refreshments	Meals provided to participants during group leader training	$2·76/person	RCT Key personnel interviews

**Table 2:** Non research cost to deliver SYV to 58 participants in intervention arm.

Categories	Item	Total units consumed	Total cost (total units consumed x unit cost)	Per participant cost (N = 58)
**Personnel**	**Non research costs**			
	Trainer salary	0·08 FTE	$543·69	$9·37
	Site supervisor salary	2·84 FTE	$12,049·30	207·75
	Group leader salary	8·50 FTE	$33,607·94	$579·45
	Subtotal	–	$46,200·93	$796·57
**Facilities**	**Non research costs**			
	Building rent for training space	2 weeks	$163·76	$2·82
	Building rent for intervention delivery space	17 months	$4,007·01	$69·09
	Subtotal	–	$4,170·77	$71·91
**Utilities**	**Non research costs**			
	Internet	74 weeks	$116·81	$2·01
	Phone calls	58 participants	$206·79	$3·57
	Subtotal	–	$323·60	$5·58
**Printing/office**				
	**Non research costs**			
	Writing utensils	17 months	$162·41	$2·80
	SYV manuals	6 group leaders	$569·64	$9·82
	Handouts	58 participants	$136·51	$2·35
	Subtotal	–	$868·56	$14·97
**Compensation**	**Non research costs**			
	Participant food vouchers	58 participants	$833·48	$14·37
	Participant travel vouchers	58 participants	$1,774·10	$30·59
	Trainer travel allowance	1 trainer	$1,780·05	$30·69
	Training snacks and refreshments	10 days	$160·20	$2·76
	Subtotal	–	$4,547·83	$78·41
	**Total**		$56,111·69	$967·44

**Table 3:** Non research cost to deliver SYV to 105 participants in intervention and control arms.

Categories	Item	Total units consumed	Total cost (total units consumed x unit cost)	Per participant cost (N = 105)
**Personnel**	**Non research costs**			
	Trainer salary	0·08 FTE	$543·69	$5·18
	Site supervisor salary	6·84 FTE	$28,185·67	$268·43
	Group leader salary	20·50 FTE	$78,723·70	$749·75
	Subtotal	–	$107,453·06	$1,023·36
**Facilities**	**Non research costs**			
	Building rent for training space	2 weeks	$163·76	$1·56
	Building rent for intervention delivery space	41 months	$9,386·07	$89·39
	Subtotal	–	$9,549·83	$90·95
**Utilities**	**Non research costs**			
	Internet	178 weeks	$278·60	$2·65
	Phone calls	105 participants	$365·78	$3·48
	Subtotal	–	$644·38	$6·13
**Printing/office**	**Non research costs**			
	Writing utensils	41 months	$382·73	$3·65
	SYV manuals	6 group leaders	$569·64	$5·43
	Handouts	105 participants	$241·47	$2·30
	Subtotal	–	$1,193·84	$11·38
**Compensation**	**Non research costs**			
	Participant food vouchers	105 participants	$1,474·31	$14·04
	Participant travel vouchers	105 participants	$3,138·14	$29·89
	Trainer travel allowance	1 trainer	$1,780·05	$16·95
	Training snacks and refreshments	10 days	$160·20	$1·53
	Subtotal	–	$6,552·70	$62·41
	**Total**		$125,393·81	$1,194·23

### Currency, price date, and conversion

We recorded program costs in Tanzanian Shillings or USD as appropriate and converted them to the 2022 USD equivalent using the consumer price index (Tables 1–3).

### Measurement and valuation of non research resources and costs

This study employed a retrospective costing approach, with data collected between May 2022 and September 2022. We recorded the costs of personnel involved in intervention training and delivery, including the SYV trainers who trained the group leaders, the group leaders, and site supervisors. We captured costs for salaried personnel in terms of annual full-time equivalents (FTEs), and multiplied unit costs by resource utilization for personnel who received wages.

Facility costs included the rent paid for the SYV group leader training and intervention delivery spaces. Utilities included internet service to the SYV office and the cost of phone calls made to participants for follow-up during intervention delivery. Printing and office supply costs included the writing utensils such as flip charts and markers used during the intervention delivery, SYV manuals, and participant handouts. Compensation costs included the travel allowance paid to the SYV trainers, the cost of light refreshments provided during group leader training, and food and travel vouchers for study participants during the intervention delivery.

Valuation of research related resources and costs is presented in Text A in S1 Appendix. These items represent expenses associated with research-related activities, such as data collection and assessing the effectiveness of the intervention (e.g., viral load and ART concentration in hair sample tests). Non research costs are detailed in the manuscript and pertain to activities and resources required for real-world implementation and scale-up of SYV, such as group leader salaries, session materials, and participant support (e.g., food and travel vouchers) (Tables 2 and 3).

### Role of funding source

The study sponsors had no role in the study design, collection, analysis, interpretation of data, writing of the report, and in the decision to submit the paper for publication.

### Ethics statement

The SYV pilot RCT study protocol was approved by the Institutional Review Boards of Kilimanjaro Christian Medical University College, the United Republic of Tanzania National Institute for Medical Research, and Duke University Health System. The approval numbers from listed review boards include the Research Ethical Clearance Certificate No.2542 from Kilimanjaro Christian Medical University College, Protocol identification number Pro00109309 from Duke University Health System, and Ethical clearance reference number NIMR/HQ/R.8a/Vol. IX/4090 from the United Republic of Tanzania National Institute for Medical Research. Participants were recruited from adolescent HIV clinics between May 10, 2016, and July 22, 2017. Written informed consent was provided by participants 18 years or older, while those under 18 years provided written assent with written permission from their caregivers.

## Results

### Study parameters

We calculated the unit costs for all variables included in our estimates and included upper and lower bounds where possible (Table 1). For study personnel, the annual salary for the two trainers who trained the SYV group leaders was US$7,060·86 per FTE. The salary paid to the group leaders who delivered the intervention over 17 months was US$3,981·00 per FTE. Group leaders’ salaries varied by their educational level. Hence, we calculated the lower bound for group leader salary as the salary of a group leader with no tertiary education as US$3,060·64 per FTE and the upper bound as the salary of a group leader with postgraduate education as US$5,541·54 per FTE. Site supervisors who managed the RCT site were paid US$4,272·12 per FTE.

The cost to rent the SYV training space was US$81·88 per week, while rent for the intervention delivery space was US$237·23 per month. Internet services cost approximately US$1·63 per week based on weekly voucher bundles utilized at the site, and the phone calls made to participants for follow-up during the intervention delivery cost approximately US$3·59 per participant. The office supplies, writing utensils, such as flip charts and markers used during the intervention delivery cost US$9·62 per month. SYV study manuals cost US$94·94 per group leader, and participant handouts cost US$2·37 per participant.

SYV study trainers were reimbursed for their travel expenses at US$1,780·00 per trainer for the two-week training of group leaders. Furthermore, light refreshments and a final meal were provided to participants during the group leader training at US$2·76 per person. Study participants received incentives in the form of food vouchers, which cost approximately US$14·47 per participant per wave (ten sessions) but ranged from US$12·85 to US$14·80. In addition, participants received travel reimbursements which cost US$30·80 per participant per wave on average but ranged from US$17·48 to US$38·32 depending on distance traveled to reach the site.

### Costs to deliver SYV to 58 participants in the intervention arm

The total cost to deliver SYV to the 58 participants randomized to the intervention arm over 41 months was US$137,618·05 and the per participant cost was US$2,372·72. The annual costs—including research-related expenses—were approximately US$58,070·61, US$49,301·58, US$20,395·98, and US$9,849·87 in the first, second, third, and fourth years respectively (see Table F in S1 Appendix). Costs incurred in the third and fourth years were related to participant follow-up, which included necessary medical tests (participant viral load and ART concentration in hair sample) and quantitative data collection.

The total non research cost of the study was US$56,111·69, non research costs per person amounted to US$967·44. Within the non research costs, personnel expenses such as group leader and site supervisor salaries comprised the largest share at 82·34% (US$46,200·93). Facilities, including the SYV training space for group leaders and intervention delivery space, accounted for 7·43% (US$4,170·77). Participant compensation (food and travel reimbursement) accounted for 8·10% (US$4,547·84) and printing and office supplies made up 1·55% (US$868·57) (Table 2).

The largest individual drivers among non research expenses were group leaders salaries (US$33,607·93), site supervisor salary (US$12,049·30), and facility costs for intervention delivery (US$4,007·01). These items accounted for 59·89%, 21·47%, and 7·14% of the total non research costs, respectively.

### Costs to deliver SYV to 105 participants in the intervention and control arms

The total cost to deliver SYV to all 105 participants randomized to the intervention arm over 58 months was US$236,597·09 and the per participant cost was US$2,253·31. The annual costs—including research-related expenses—were approximately US$51,328·20, US$64,434·68, US$60,931·66, US$42,376·55, and US$17,526·02 in the first through fifth years respectively (see Table G in S1 Appendix).

The total non research costs of the study was US$125,393·80, non research costs per person amounted to US$1,194·23. Within the non research costs, personnel expenses such as group leader and site supervisor salaries comprised the largest share at 85·69% (US$107,453·06). Facilities, including the SYV training space for group leaders and intervention delivery space, accounted for 7·62% (US$9,549·93). Participant compensation (food and travel reimbursement) accounted for 5·23% (US$6,552·70) and printing and office supplies made up 0·95% (US$1,193·84) (Table 3).

The largest individual drivers among non research expenses were group leaders salaries (US$78,723·69), site supervisor salary (US$28,185·69), and facility costs for intervention delivery (US$9,386·07). These items accounted for 62·78%, 22·48%, and 7·49% of the total non research costs, respectively.

### Sensitivity analysis

**Fig 2:**
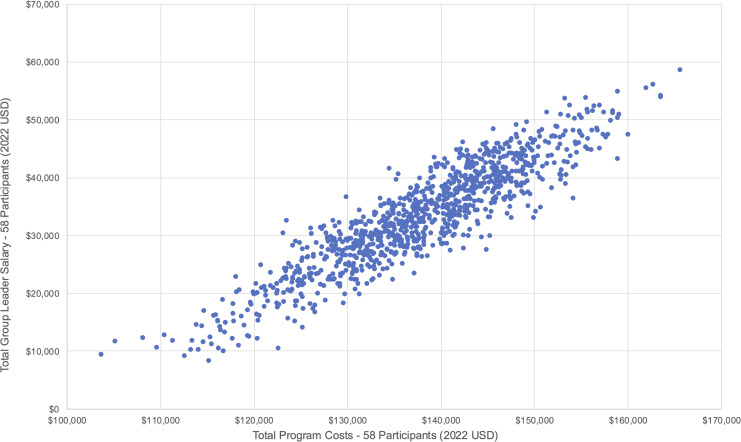
Probabilistic sensitivity analyses – Group leader salary (Scenario 1 – 58 participants).

**Fig 3:**
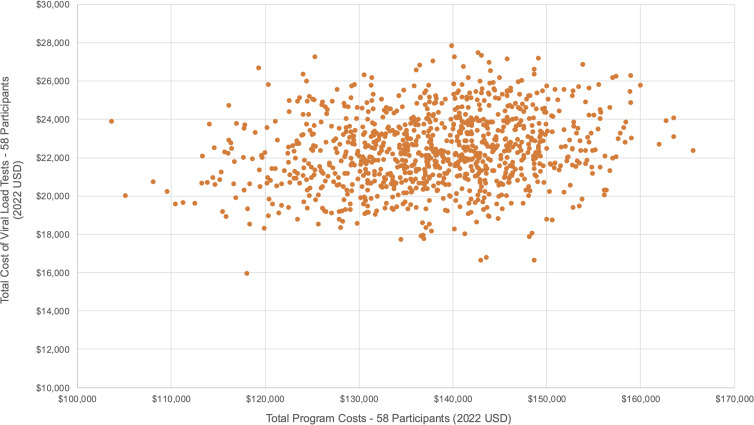
Probabilistic sensitivity analysis – Viral load tests (Scenario 1 – 58 participants).

**Fig 4:**
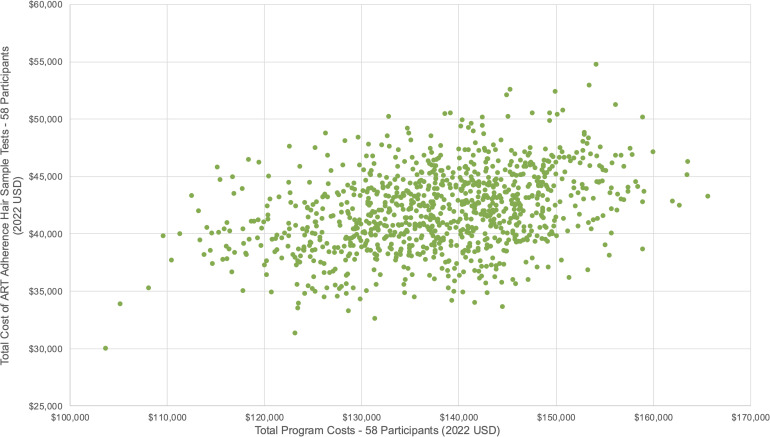
Probabilistic sensitivity analysis – Hair sample adherence tests (Scenario 1 – 58 participants).

We conducted a probabilistic sensitivity analysis to account for uncertainties in our cost estimates. We varied unit estimates of the group leader’s salary, viral load tests, ART adherence tests, travel vouchers, and food vouchers around their respective means, upper, and lower bounds (see Fig 2–3, and Fig 4). Although viral load and ART adherence tests were research related, they were significant cost drivers of total costs and therefore included in the sensitivity analysis. From our results, the total cost to deliver SYV to 58 participants was US$137,835·72 [95% CI: $137,722·98, $138,448·46], and the total per participant cost was US$2,368·99 [95% CI: $2,358·43, $2,379·55]. Non research costs amounted to US$54,286·70 [95% CI: $53,729·76, $54,843·63], and the total non research cost per participant was US$935·98 [95% CI: $926·38, $945·58].

**Fig 5:**
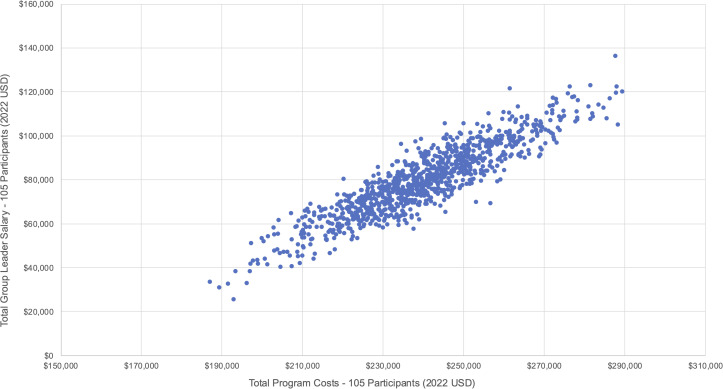
Probabilistic sensitivity analyses – Group leader salary (Scenario 2 – 105 participants).

**Fig 6:**
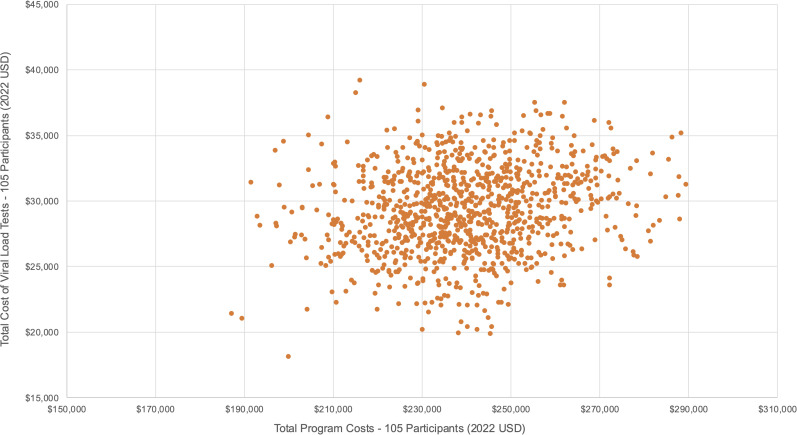
Probabilistic sensitivity analysis – Viral load tests (Scenario 2 – 105 participants).

**Fig 7:**
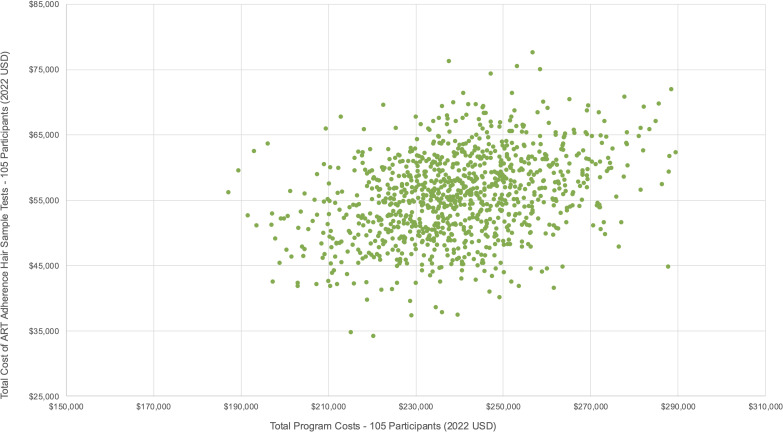
Probabilistic sensitivity analysis – Hair sample adherence tests (Scenario 2 – 105 participants).

For the second scenario, the cost to deliver SYV to 105 participants was US$239,835·95 [95% CI: $238,760·81, $240,911·08], and the cost per participant was US$2,284·15 [95% CI: $2,273·91, $2,294·39] (see Fig 5–6, and Fig 7). Non research costs amounted to US$127,705·01, [95% CI: $126,716·18, $128,693·83] and the total non research cost per participant was US$1,216·24 [95% CI: $1,206·82, $1,225·66]. We provide more details of our sensitivity analyses in the appendix (see Tables H and I in S1 Appendix).

In addition, we explored the total costs of SYV delivery discounted at an annual rate of 3% for both scenarios. The total discounted cost of SYV delivery to 58 participants in Scenario 1 was approximately US$134,175·42. In Scenario 2, the total discounted cost of the SYV program delivered to all 105 participants was approximately US$225,672·26.

## Discussion

Our results show that total and per participant costs of delivering SYV to 58 patients in the context of a pilot RCT conducted in the city of Moshi were US$137,835·72 [95% CI: $137,722·98, $138,448·46] and US$2,368·99 [95% CI: $2,358·43, $2,379·55] respectively. We performed probabilistic sensitivity analyses by varying the lower, mean, and upper bounds of key unit costs—including the group leader’s salary, viral load and adherence tests, travel vouchers, and food vouchers. The changes in total and per-participant costs were modest across both scenarios and overall costs remained relatively stable. Although these variations were modest in our study, they could have a greater impact on scale-up of the intervention. To our knowledge, this is the first study to estimate the costs of delivering a mental health intervention to YPLWH in Tanzania. These figures provide a valuable benchmark for the expansion of SYV across Tanzania, offering a pioneering dataset for estimating costs in larger-scale implementations.

Our results demonstrate that SYV, being a comprehensive and research-intensive intervention, tends to be more costly than other interventions designed to improve ART adherence among YPLWH in the sub-Saharan African context. For example, the total non-research cost of delivering six HIV and ART adherence counseling sessions provided by lay counsellors and peer navigators in addition to economic security workshops to 358 HIV-positive youth between the ages of 10–16 years in Uganda in 2015 was US$263·00 (approximately US$316·00 in 2022) per participant reached over two years. Furthermore, the Ugandan intervention resulted in an 8·85 percentage point increase in the proportion of virally suppressed (HIV RNA < 40 copies/mL) adolescents between participants who received both ART adherence counseling sessions and economic security workshops to participants who received only ART adherence counseling sessions [21]. For comparison, the SYV RCT previously reported a ten percentage point increase in viral suppression rates (HIV RNA < 400 copies/mL) between the SYV and SOC arms (RR 1·13 [95% CI: 0·94, 1·36]) which was not statistically significant [18]. In our analysis, achieving this previously reported improvement required an incremental non research cost of US$967·44 per participant over the program’s four-year duration.

As another example, the cost of delivering a community-based support (CBS) intervention designed to improve ART adherence among HIV-positive youth aged 10–24 years and delivered by lay community workers in South Africa in 2012 was US$49·50 (approximately US$61·40 in 2022) per participant per year. The CBS intervention involved home-based ART-related education, pyscho social support, screening for opportunistic infections, and assistance with accessing government grants. Results from the CBS intervention showed it had no effect on viral suppression rates in CBS participants compared to non-CBS participants after three years. However, after five years, there was an 18·40 percentage point difference in viral suppression rates between CBS and non-CBS participants [22]. In contrast, SYV cost estimates are similar to estimates from the Zvandiri intervention for adolescents living with HIV in Zimbabwe. In the Zvandiri model, mental health support was delivered to adolescents living with HIV through community adolescent treatment supporters (CATS) and included monthly support group meetings, home visits, caregiver workshops for guardians and family member. The intervention was estimated to cost US$997·00 per ART client per year and US$1,340·00 per adolescent virally suppressed per year from a health provider perspective. The Zvandiri cost analysis included capital expenditures such as buildings and storage costs and equipment, as well as recurrent costs such as personnel, drug supplies, and laboratory supplies from the intervention clinics [23]. While these comparisons highlight the distinct costs and impact of SYV compared to similar interventions for YPLWH, they warrant cautious interpretation due to significant differences in the program structure, study design, health system context, and cost metrics.

Several factors may account for the higher cost of SYV delivery compared to other interventions designed to improve ART adherence among YPLWH. These factors include the cost of the research components in the SYV program that would not be included in routine care, e.g., ART adherence levels established through testing hair samples, collecting blood samples and performing viral load assays, which in the case of SYV were done more frequently than for routine care. Additionally, six group leaders delivered the intervention to 105 participants over six waves, with approximately 18 participants per wave leading to a participant-to-group leader ratio of 3:1 per wave. The factors listed above were significant cost drivers of the SYV RCT. Although costly compared to some previously reported work in neighboring countries, costs alone are insufficient to gauge the value of scaling up SYV to reach YPLWH across Tanzania. While outside the scope of this study, a cost-effectiveness analysis is ultimately needed to examine whether the health and economic benefits of scaling up SYV across Tanzania outweigh the required investments. With the current adolescent mental health crisis and the critical mental health worker shortage (0·52 health workers per 100,000 population), SYV goes beyond adherence to provide youth with coping strategies that help address and prevent mental distress, and may have critical impact on future economic contribution to society [24].

Nevertheless, our findings carry significant policy implications. In 2021, UNAIDS published its updated 95-95-95 strategy to achieve a global target of 95% of all people living with HIV diagnosed, 95% of all people diagnosed with HIV on ART, and 95% of all people with HIV on ART virally suppressed [25]. In alignment with this, Tanzania’s National AIDS Control Programme (NACP) developed its fourth Health Sector HIV and AIDS Strategic Plan (HSHSP IV) for 2017–2022 [26]. The HSHSP IV was informed in part by evidence of increasing new HIV infections among young people and emphasizes the need to improve both ART adherence among adolescents and the availability of ART adherence support services across the country. To further improve Tanzania’s response to increasing HIV infections among young people and to meet the UNAIDS target, the Ministry of Health, together with the NACP, published a National Training Package on Adolescents Living with HIV and AIDS in 2017 to help health workers acquire mental health literacy and skills necessary to provide quality care and counselling to infected adolescents [27,28]. This initiative reflects Tanzania’s intensified focus on adolescents. However, challenges persist as the 2023 Tanzania HIV Impact Survey reveals a disparity in viral suppression rates across different age groups with only 59·50% of YPLWH virally suppressed, compared to 78% in those aged 15 and older[ [29]. While further research is needed to guide the implementation and scale-up of novel interventions that address the syndemic of mental distress and HIV among young people, our study provides valuable insights on the costs of interventions critical to achieving the UNAIDS targets.

The strengths of our paper include the use of a hybrid-costing approach. Where possible, we employed a micro-costing methodology, which produces precise cost estimates by carefully identifying resource utilization and unit costs [30]. Furthermore, good recordkeeping by the SYV program granted us access to robust financial and resource utilization data. Limitations of the study include the potential for recall bias in data collected from RCT key personnel, as the SYV pilot RCT was conducted between 2016–2020, while the cost analysis took place two years later in 2022. Additionally, we use an intent-to-treat (ITT) approach where we assume that the intervention was delivered to all enrolled participants in the trial (58 SYV participants in scenario 1 and 105 SYV and SOC participants in scenario 2). We also assume that all participants were followed up at all data collection time points (Baseline, 6 months, 12 months, 18 months, and 30 months for SYV participants, and baseline and 6 months for SOC participants in the crossover waves). This approach may significantly overestimate the actual costs of the SYV RCT.

## Conclusion

This study provides a cost analysis of delivering a mental health intervention aimed at improving ART adherence and viral suppression among YPLWH in Tanzania, based on its implementation in a pilot RCT. Our analysis suggests that SYV is financially feasible to scale, particularly since over half of its total program costs were attributable to research-related activities that are not required in routine implementation. However, a cost-effectiveness analysis will be needed to further assess the intervention’s value relative to its health outcomes. These cost estimates are vital for informing current and future strategies to combat the HIV epidemic among adolescents by establishing a benchmark for the implementation of mental health interventions targeting YPLWH in the sub-Saharan context. Given the global drive to meet the UNAIDS 95-95-95 HIV targets, tailoring interventions for specific sub-groups, such as adolescents aged 10–24 experiencing increased HIV infection rates and lower viral suppression levels, is crucial to achieving these goals.

## Supporting information

S1 Appendix Text A. Measurement and valuation of resources and costs. (DOCX)

S1 File Checklist. (DOCX)
