# Supplementary material for: Cost of Sauti ya Vijana (SYV), a mental health intervention for young people living with HIV in Tanzania: Results from a pilot randomized controlled trial
Source: PLOS Glob Public Health. 2025 Dec 18;5(12):e0005397. doi: 10.1371/journal.pgph.0005397 (PMC12714259; doi:10.1371/journal.pgph.0005397)
Supplement: S1 Appendix — (DOCX) [file pgph.0005397.s001.docx]

**S1 Appendix. Supporting information**

**Text A. Measurement and valuation of resources and costs**

**Personnel costs**

Personnel costs included salaries paid to the individuals who trained the SYV group leaders, salaries paid to the SYV group leaders who delivered the intervention, fees paid to the research assistants who conducted the participant interviews and questionnaires, fees paid to the phlebotomists who collected participant blood samples, salaries paid to the supervisor overseeing delivery of the intervention at the research site, and fees paid to individuals hired to transcribe and translate all qualitative data that was collected. Salary resource utilization was measured in terms of full-time equivalents (FTEs), where one FTE represents 52 weeks (50 weeks of work + two weeks of paid time off).

**Facility and utility costs**

Facility and utility costs included rent for the venue used to train the group leaders, rent for the venue used to deliver the intervention, internet service, and phone service used to make follow-up calls to participants after each intervention session.

**Printing and office supply costs**

Printing and office supply costs included writing utensils needed to deliver the intervention, printing charges for manuals designed to help group leaders deliver the intervention, printing charges for questionnaires delivered to participants, and printing charges for participant handouts.

**Laboratory testing costs**

Testing costs included viral load tests conducted on participant blood samples collected at each data collection point as well as ART adherence tests conducted on participant hair samples collected at each data collection point.

**Compensatory costs**

Compensatory costs included food and travel vouchers given to participants at each intervention session as reimbursement for meal and transportation expenses incurred for attending the intervention. Compensatory costs also included an allowance given to the group leader trainers for travel to and from the training site.

**Miscellaneous costs**

Miscellaneous costs included National Institute of Medical Research and IRB fees for each year that the pilot RCT was conducted as well as administrative fees for the study. Eight percent of the pilot RCT budget was allocated for administrative expenses for each scenario modeled. we, therefore, calculate administrative expenses as 8·0% of the total costs across all items/activities included in the scenario, which is the standard fee charged at the Kilimanjaro Christian Medical Centre, Moshi, Tanzania.

**Research related costs**

We include research-related costs in our analysis. Research-related costs are those costs pertaining to any items that were necessary to determine the efficacy of the SYV intervention in the pilot RCT. These items include IRB renewal fees, research assistant fees, printing charges for the participant questionnaires and handouts, transcription and translation fees, phlebotomist fees, viral load tests, and ART adherence tests. We expect that routine implementation of SYV in an HIV clinic would not require these research-related items.

**Participant data collection visit schedule and costing assumptions**

The visit schedule was standardized across arms during the main trial period (baseline, 6, 12, 18, and 30 months) for both SYV and SOC participants. In the cross-over design, the SOC group later received SYV and was evaluated at two additional points (baseline and post-intervention). This costing analysis included only the periods when participants received SYV (including SOC participants once they crossed over), focusing on the incremental cost of delivering the intervention. Thus, the higher number of visits in the SYV arm (290 study visits) compared to the SOC arm (94 study visits) reflects the trial design and does not artificially inflate intervention costs.

**All study parameters**

We calculated the unit costs for all variables included in our estimates and included upper and lower bounds where possible (Table 1). For study personnel, the annual salary for the two trainers who trained the SYV group leaders was US$7,060·86 per FTE. The salary paid to the group leaders who delivered the intervention over 17 months was US$3,981·00 per FTE. Group leaders’ salaries varied by their educational level. Hence, we calculated the lower bound for group leader salary as the salary of a group leader with no tertiary education as US$3,060·64 per FTE and the upper bound as the salary of a group leader with postgraduate education as US$5,541·54 per FTE. Research assistants who conducted participant interviews were paid a stipend of US$10·68 per interview, and phlebotomists were paid approximately US$1·78 per sample collected. Site supervisors who managed the RCT site were paid US$4,272·12 per FTE. Transcription/translation fees amounted to approximately US$12·29 per month.

The cost to rent the SYV training space was US$81·88 per week, while rent for the intervention delivery space was US$237·23 per month. Internet services cost approximately US$1·63 per week based on weekly voucher bundles utilized at the site, and the phone calls made to participants for follow-up during the intervention delivery cost approximately US$3·59 per participant. For the office supplies, writing utensils, such as flip charts and markers used during the intervention delivery, cost US$9·62 per month. SYV study manuals cost US$94·94 per group leader, questionnaires used for participant interviews cost US$5·93 per participant, and participant handouts cost US$2·37 per participant. For study tests, participant viral load tests cost US$79·47 per test, and hair sample ART adherence tests cost US$149·52 per test. In our probabilistic sensitivity analysis, we varied the cost of viral load tests and ART adherence tests by 10%.

SYV study trainers were reimbursed for their travel expenses at US$1,780·00 per trainer for the two-week training of group leaders. Furthermore, light refreshments and a final meal were provided to participants during the group leader training at US$2·76 per person. Study participants received incentives in the form of food vouchers, which cost approximately US$14·47 per participant per wave (ten sessions) but ranged from US$12·85 to US$14·80. In addition, participants received travel reimbursements which cost US$30·80 per participant per wave on average but ranged from US$17·48 to US$38·32 depending on distance traveled to reach the site. KCMC institutional review board fees cost US$125·01 per annum, while the National Institute of Medical Research fees were US$500·00 dollars for the first year and US$100·00 for subsequent years for renewals.

**Sensitivity analysis**

We conducted a multi-way probabilistic sensitivity analysis on scenarios 1 and 2 to account for uncertainty in our unit cost estimates. We ran 1,000 simulations, randomly sampling parameter values for group leader salary, viral load test, ART adherence test, food voucher, and travel voucher costs from their respective normal distributions. The parameters and their ranges were chosen based on observed variation in pilot data or plausible market fluctuations.

The unit cost for group leaders’ salary was based on the average salary paid to the group leaders who delivered the SYV intervention in the pilot RCT. Group leaders were paid based on their level of education. Therefore, for the sensitivity analysis, we model a lower bound unit cost that reflects the monthly salaries paid to group leaders with a high school education only and an upper bound unit cost that reflects the monthly salaries paid to group leaders with a university degree. For viral load and ART adherence tests, we model a 10% reduction and increase in the unit cost.

The total program costs and individual item costs were averaged across the 1,000 simulations to obtain mean costs with 95% confidence intervals.

Table A. Discounted cost of SYV delivery over time (3% discount rate) – Scenario 1 (58 Participants)

| **Year** | **Costs** | **Costs discounted at 3%** |
| --- | --- | --- |
| 1 | $58,070·61 | $58,070·61 |
| 2 | $49,301·58 | $47,865·61 |
| 3 | $20,395·98 | $19,225·17 |
| 4 | $9,948·87 | $9,014·03 |
| **Total** | $**137,618**·**04** | $**134,175**·4**2** |

Table B. Discounted cost of SYV delivery over time (3% discount rate) – Scenario 2 (105 Participants)

| **Year** | **Costs** | **Costs discounted at 3%** |
| --- | --- | --- |
| 1 | $51,328·20 | $51,328·20 |
| 2 | $64,434·68 | $62,557·94 |
| 3 | $60,931·66 | $57,433·93 |
| 4 | $42,376·55 | $38,780·54 |
| 5 | $17,526·02 | $15,571·64 |
|  | $**236,597**·**10** | **$225,672·26** |

Table C. Costing parameters

| **Item** | **Item definition** | **Cost/unit** | **Data source** |
| --- | --- | --- | --- |
| **Personnel** |  |  |  |
| Trainer salary | Salary paid to individuals who trained the group leaders | $7,060·86/FTE | RCT Key personnel interviews |
| Group leader salary | Salary paid to group leaders who delivered the intervention | $3,981·28/FTE | RCT financial records |
| Research assistant fees | Fees paid to research assistants to conduct participant interviews and questionnaires | $10·68/interview | RCT financial records |
| Phlebotomist fees | Fees paid to phlebotomists for blood sample collection | $1·78/blood sample | RCT financial records |
| Site supervisor salary | Salary paid to supervisor overseeing research site | $4,272·12/FTE | RCT Key personnel interviews |
| Transcription/translation fees | Fees paid to transcribe and translate documents | $12·29/month | RCT financial records |
| **Facilities** |  |  |  |
| Building rent for training space | Rent for facility space used to conduct group leader trainings | $81·88/week | RCT Key personnel interviews |
| Building rent for intervention delivery space | Rent for facility space used to deliver the intervention | $237·34/month | RCT financial records |
| **Utilities** |  |  |  |
| Internet | Internet service | $1·63/week | RCT Key personnel interviews |
| Phone calls | Follow-up phone calls to participants | $3·59/participant | RCT financial records and key personnel interviews |
| **Printing/office** |  |  |  |
| Writing utensils | Writing materials used to deliver the intervention | $9·62/month | RCT financial records |
| SYV manuals | Manuals to facilitate group leader trainings | $94·94/group leader | RCT Key personnel interviews |
| Questionnaires | Participant questionnaires | $5·93/questionnaire | RCT Key personnel interviews |
| Handouts | Participant handouts | $2·37/participant | RCT Key personnel interviews |
| **Testing** |  |  |  |
| Viral load tests | Participant viral load tests | $79·47/test | RCT financial records |
| ART adherence tests | Participant ART adherence tests | $149·52/test | RCT financial records |
| **Compensation** |  |  |  |
| Participant food vouchers | Food vouchers given to participants per SYV intervention wave | $14·47/participant | RCT financial records |
| Participant travel vouchers | Travel vouchers given to participants per SYV intervention wave | $30·80/participant | RCT financial records |
| Trainer travel allowance | Allowance provided for a U.S.-based trainer traveling to conduct training for group leaders | $1,780·00/trainer | RCT financial records |
| Training snacks and refreshments | Meals provided to participants during group leader training | $2·76/person | RCT Key personnel interviews |
| **Miscellaneous** |  |  |  |
| IRB fees | Fees for annual IRB processing and renewal | $125·01/year | RCT financial records |
| National Institute of Medical Research Fees | Fees for annual processing and renewal | $500·00 for first year, $100·00 for subsequent years | RCT Key personnel interviews |

Table D. Cost to deliver SYV – Scenario 1 (58 participants)

| **Categories** | **Item** | **Total units consumed** | **Total cost (total units consumed x unit cost)** | **Per participant cost (N=58)** |
| --- | --- | --- | --- | --- |
| **Personnel** | **Research costs** | | | |
|  | Phlebotomist fees | 290 blood samples | $501·62 | $8·65 |
|  | Research assistant fees | 290 interviews | $3,009·75 | $51·89 |
|  | Transcription/translation fees | 17 months | $208·93 | $3·60 |
|  | Subtotal (research costs) |  | $3,720·30 | $64·14 |
|  |  | | | |
|  | **Non research costs** | | | |
|  | Trainer salary | 0·08 FTE | $543·69 | $9·37 |
|  | Site supervisor salary | 2·84 FTE | $12,049·30 | 207·75 |
|  | Group leader salary | 8·50 FTE | $33,607·94 | $579·45 |
|  | Subtotal (Non research costs) | - | $46,200·93 | $796·57 |
| **Facilities** | **Non research costs** | | |  |
|  | Building rent for training space | 2 weeks | $163·76 | $2·82 |
|  | Building rent for intervention delivery space | 17 months | $4,007·01 | $69·09 |
|  | Subtotal (Non research costs) | - | $4,170·77 | $71·91 |
| **Utilities** | **Non research costs** | | | |
|  | Internet | 74 weeks | $116·81 | $2·01 |
|  | Phone calls | 58 participants | $206·79 | $3·57 |
|  | Subtotal (Non research costs) | - | $323·60 | $5·58 |
| **Printing/office** | **Research costs** | | | |
|  | Questionnaires | 290 questionnaires | $1,671·14 | $28·81 |
|  | Subtotal (research costs) |  | $1,671·14 | $28·81 |
|  |  |  |  |  |
|  | **Non research costs** |  |  |  |
|  | Writing utensils | 17 months | $162·41 | $2·80 |
|  | SYV manuals | 6 group leaders | $569·64 | $9·82 |
|  | Handouts | 58 participants | $136·51 | $2·35 |
|  | Subtotal (Non research costs) | - | $868·56 | $14·97 |
| **Testing** | **Research costs** |  |  |  |
|  | Viral load (HIV RNA) | 290 tests | $22,395·55 | $386·13 |
|  | ART adherence tests | 290 tests | $42,136·43 | $726·49 |
|  | Subtotal (research costs) | - | $64,531·98 | $1,112·62 |
| **Compensation** | **Non research costs** | | | |
|  | Participant food vouchers | 58 participants | $833·48 | $14·37 |
|  | Participant travel vouchers | 58 participants | $1,774·10 | $30·59 |
|  | Trainer travel allowance | 1 trainer | $1,780·05 | $30·69 |
|  | Training snacks and refreshments | 10 days | $160·20 | $2·76 |
|  | Subtotal (Non research costs) | - | $4,547·83 | $78·41 |
| **Miscellaneous** | **Research costs** | | | |
|  | IRB fees | 4 years | $484·21 | $8·34 |
|  | National Institute of Medical Research Fees | 4 years | $904·79 | $15·59 |
|  | Administrative fees | NA | $10,193·93 | $175·76 |
|  | Subtotal (research costs) | - | $11,582·93 | $199·69 |
|  | **Total** |  | $137,618·05 | $2,372·72 |

Table E. Cost to deliver SYV – Scenario 2 (105 participants)

| **Categories** | **Item** | **Total units consumed** | **Total cost (total units consumed x unit cost)** | **Per participant cost (N=105)** |
| --- | --- | --- | --- | --- |
| **Personnel** | **Research costs** | | | |
|  | Phlebotomist fees | 384 blood samples | $660·50 | $6·29 |
|  | Research assistant fees | 384 interviews | $3,958·16 | $37·70 |
|  | Transcription/translation fees | 41 months | $503·89 | $4·80 |
|  | Subtotal (research costs) |  | $5,122·55 | $48·79 |
|  | **Non research costs** |  |  |  |
|  | Trainer salary | 0·08 FTE | $543·69 | $5·18 |
|  | Site supervisor salary | 6·84 FTE | $28,185·67 | $268·43 |
|  | Group leader salary | 20·50 FTE | $78,723·70 | $749·75 |
|  | Subtotal (non research costs) | - | $107,453·06 | $1,023·36 |
| **Facilities** | **Non research costs** | | | |
|  | Building rent for training space | 2 weeks | $163·76 | $1·56 |
|  | Building rent for intervention delivery space | 41 months | $9,386·07 | $89·39 |
|  | Subtotal (non research costs) | - | $9,549·83 | $90·95 |
| **Utilities** | **Non research costs** | | | |
|  | Internet | 178 weeks | $278·60 | $2·65 |
|  | Phone calls | 105 participants | $365·78 | $3·48 |
|  | Subtotal (non research costs) | - | $644·38 | $6·13 |
| **Printing/office** | **Research costs** | | | |
|  | Questionnaires | 384 questionnaires | $2,200·44 | $20·96 |
|  | Subtotal (research costs) |  | $2,200·44 | $20·96 |
|  | **Non research costs** |  |  |  |
|  | Writing utensils | 41 months | $382·73 | $3·65 |
|  | SYV manuals | 6 group leaders | $569·64 | $5·43 |
|  | Handouts | 105 participants | $241·47 | $2·30 |
|  | Subtotal (non research costs) | - | $1,193·84 | $11·38 |
| **Testing** | **Research costs** | | | |
|  | Viral load (HIV RNA) | 384 tests | $29,499·87 | $280·85 |
|  | ART adherence tests | 384 tests | $55,482·28 | $528·40 |
|  | Subtotal (research costs) | - | $84,982·15 | $809·25 |
| **Compensation** | **Non research costs** | | | |
|  | Participant food vouchers | 105 participants | $1,474·31 | $14·04 |
|  | Participant travel vouchers | 105 participants | $3,138·14 | $29·89 |
|  | Trainer travel allowance | 1 trainer | $1,780·05 | $16·95 |
|  | Training snacks and refreshments | 10 days | $160·20 | $1·53 |
|  | Subtotal (non research costs) | - | $6,552·70 | $62·41 |
| **Miscellaneous** | **Research costs** | | | |
|  | IRB fees | 4 years | $482·26 | $4·59 |
|  | National Institute of Medical Research Fees | 4 years | $901·17 | $8·58 |
|  | Administrative fees | NA | $17,525·71 | $166·91 |
|  | Subtotal (research costs) | - | $18,909·14 | $180·08 |
|  | **Total** |  | $236,597·09 | $2,253·31 |

Table F. Annual inflation-adjusted breakdown of SYV delivery costs and resource use – Scenario 1 (58 Participants)

| **Category** | **Research/non research costs** | **Item** | **Year 1** | | **Year 2** | | **Year 3** | | **Year 4** | |
| --- | --- | --- | --- | --- | --- | --- | --- | --- | --- | --- |
|  |  |  | **Resource use** | **Costs (USD)** | **Resource use** | **Costs (USD)** | **Resource use** | **Costs (USD)** | **Resource use** | **Costs (USD)** |
| Compensation | Non research | Training snacks and refreshments | 10 days | 160·20 | - | - | - | - | - | - |
| Personnel | Non research | Trainer salary | 0·08 FTE | 543·69 | - | - | - | - | - | - |
| Compensation | Non research | Trainer travel allowance | 1 trainer | 1,780·05 | - | - | - | - | - | - |
| Facilities | Non research | Training facility space | 14 days | 163·76 | - | - | - | - | - | - |
| Personnel | Research | Translation services | 1 year | 208·93 | - | - | - | - | - | - |
| Testing | Research | ART adherence tests | 58 tests | 8,672·16 | 116 tests | 16,982·61 | 78 tests | 11,189·95 | 38 tests | 5,291·87 |
| Personnel | Non research | Site supervisor salary | 4·58 FTE | 8,128·99 | 2·26 FTE | 3,920·31 | - |  | - |  |
| Miscellaneous | Research | Institutional Review Board + National Institute for Medical Research fees (Average across years) | 1 year | 358·57 | 1 year | 351·09 | 1 year | 342·72 | 1 year | 336·62 |
| Personnel | Non research | Group leader salary | 5·70 FTE | 22,673·39 | 2·80 FTE | 10,934·55 | - |  | - |  |
| Facilities | Non research | Facility space | 1 year | 2,703·30 | <1 year | 1,303·71 | - |  | - |  |
| Personnel | Research | Research assistant fee | 58 interviews | 619·44 | 116 interviews | 1,213·04 | 78 interviews | 799·27 | 38 interviews | 377·99 |
| Personnel | Research | Phlebotomist fees | 58 samples | 103·24 | 116 samples | 202·17 | 78 samples | 133·21 | 38 samples | 63·00 |
| Testing | Research | Laboratory tests | 58 tests | 4,609·26 | 116 tests | 9,026·23 | 78 tests | 5,947·44 | 38 tests | 2,812·62 |
| Compensation | Non research | Food vouchers for patients | 39 vouchers | 562·30 | 19 vouchers | 271·18 | - |  | - |  |
| Compensation | Non research | Travel vouchers for patients | 39 vouchers | 1,196·89 | 19 vouchers | 577·21 | - |  | - |  |
| Utilities | Non research | Internet services | 1 year | 30·16 | 1 year | 29·53 | 1 year | 28·83 | 1 year | 28·31 |
| Utilities | Non research | Phone calls | 39 participants | 139·51 | 19 participants | 67·28 | - |  | - |  |
| Printing/Office | Non research | Writing utensils | 12 months | 109·57 | 6 months | 52·84 | - |  | - |  |
| Printing/Office | Non research | SYV manuals | 6 manuals | 569·64 | - |  | - |  | - |  |
| Printing/Office | Non research | Questionnaires | 58 Questionnaires | 343·94 | 116 Questionnaires | 673·53 | 78 Questionnaires | 443·80 | 38 Questionnaires | 209·87 |
| Printing/Office | Non research | Participant handouts | 39 participants | 92·10 | 19 participants | 44·65 | - |  | - |  |
| Miscellaneous | Non research | Administrative fees | 1 year | 4,301·53 | 1 year | 3,651·98 | 1 year | 1,510·82 | 1 year | 729·62 |
| Total | | | | 58,070·61 |  | 49,301·67 |  | 20,396·03 |  | 9,849·90 |

Table G. Annual inflation-adjusted breakdown of SYV delivery costs and resource use – Scenario 2 (105 Participants)

| **Category** | **Research/non research costs** | **Item** | **Year 1** | | **Year 2** | | **Year 3** | | **Year 4** | | **Year 5** | |
| --- | --- | --- | --- | --- | --- | --- | --- | --- | --- | --- | --- | --- |
|  |  |  | **Resource use** | **Costs (USD)** | **Resource use** | **Costs (USD)** | **Resource use** | **Costs (USD)** | **Resource use** | **Costs (USD)** | **Resource use** | **Costs (USD)** |
| Compensation | Non research | Training snacks and refreshments | 10 days | 160·20 | - | - | - | - | - | - | - | - |
| Personnel | Non research | Trainer salary | 0·08 FTE | 543·69 | - | - | - | - | - | - | - | - |
| Compensation | Non research | Trainer travel allowance | 1 trainer | 1,780·05 | - | - | - | - | - | - | - | - |
| Facilities | Non research | Training facility space | 14 days | 163·76 | - | - | - | - | - | - | - | - |
| Personnel | Research | Translation services | 1 year | 503·89 | - | - | - | - | - | - | - | - |
| Testing | Research | ART adherence tests | 58 tests | 8,612·35 | 127 tests | 18,551·95 | 127 tests | 18,109·62 | 54 tests | 7,546·14 | 19 tests | 2,662·20 |
| Personnel | Non research | Site supervisor salary | 1·52 FTE | 6,618·62 | 1·52 FTE | 6,477·70 | 1·52 FTE | 6,323·26 | 1·52 FTE | 6,210·72 | 0·76 FTE | 2,555·36 |
| Miscellaneous | Research | Institutional Review Board + National Institute for Medical Research fees (Average across years) | 1 year | 324·74 | 1 year | 317·97 | 1 year | 310·39 | 1 year | 304·86 | 1 year | 125·48 |
| Personnel | Non research | Group leader salary | 4·56 FTE | 18,486·08 | 4·56 FTE | 18,092·49 | 4·56 FTE | 17,661·12 | 4·56 FTE | - | 2·28 FTE | 7,137·22 |
| Facilities | Non research | Facility space | 1 year | 2,204·06 | 1 year | 2,157·13 | 1 year | 2,105·69 | 1 year | 2,068·22 | 5 months | 850·96 |
| Personnel | Research | Research assistant fee | 54 interviews | 574·16 | 127 interviews | 1,325·14 | 127 interviews | 1,293·55 | 38 interviews | 385·01 | 38 interviews | 380·31 |
| Personnel | Research | Phlebotomist fees | 58 samples | 102·53 | 127 samples | 220·86 | 127 samples | 215·59 | 54 samples | 89·83 | 19 samples | 31·70 |
| Testing | Research | Laboratory tests | 58 tests | 4,577·47 | 127 tests | 9,860·38 | 127 tests | 9,625·28 | 54 tests | 4,010·78 | 19 tests | 1,414·96 |
| Compensation | Non research | Food vouchers for patients | 35 vouchers | 501·39 | 35 vouchers | 490·93 | - | - | 18 vouchers | 242·48 | 18 vouchers | 239·52 |
| Compensation | Non research | Travel vouchers for patients | 35 vouchers | 1,067·22 | 35 vouchers | 1,044·96 | - | - | 18 vouchers | 516·13 | 18 vouchers | 509·84 |
| Utilities | Non research | Internet services | 1 year | 58·03 | 1 year | 56·82 | 1 year | 55·46 | 1 year | 54·48 | 1 year | 53·81 |
| Utilities | Non research | Phone calls | 35 participants | 124·39 | 35 participants | 121·80 | - | - | 18 participants | 60·16 | 17 participants | 59·42 |
| Printing/Office | Non research | Writing utensils | 12 months | 130·16 | 12 months | 127·44 | - | - | 8 months | 62·95 | 8 months | 62·18 |
| Printing/Office | Non research | SYV manuals | 6 manuals | 569·64 | - | - | - | - | - | - | - | - |
| Printing/Office | Non research | Questionnaires | 58 questionnaires | 341·57 | 127 questionnaires | 735·78 | 127 questionnaires | 718·23 | 54 questionnaires | 299·28 | 19 questionnaires | 105·59 |
| Printing/Office | Non research | Participant handouts | 35 participants | 82·12 | 35 participants | 80·41 | - | - | 18 participants | 39·71 | 18 participants | 39·23 |
| Miscellaneous | Non research | Administrative fees | 1 year | 3,802·46 | 1 year | - | 1 year | 4,513·81 | 1 year | 3,139·35 | 1 year | 1,298·57 |
| Total | | | | 51,328·20 |  | 64,434·67 |  | 60,931·65 |  | 42,376·55 |  | 17,526·03 |

Table H. Sensitivity analyses – Scenario 1 (58 Participants)

| **Item** | **Mean** | **95% Confidence interval** |
| --- | --- | --- |
| Group leader salary | $33,441·59 | $32,886·42 - $33,996·76 |
| Viral load tests | $22,391·27 | $22,272·43 - $22,510·10 |
| ART adherence tests | $42,012·20 | $41,796·72 - $42,227·69 |
| Travel vouchers | $1,778·71 | $1,747·40 - $1,810·02 |
| Food vouchers | $831·67 | $821·08 - $842·26 |
| Total (All variables) | $137,835·72 | $137,722·98 - $138,448·46 |
| Total per participant (All variables) | $2,368·99 | $2,358·43 - $2,379·55 |

Table I. Sensitivity analyses – Scenario 2 (105 Participants)

| **Item** | **Mean** | **95% Confidence interval** |
| --- | --- | --- |
| Group leader salary | $79,615·60 | $78,626·41 - $80,604·79 |
| Viral load tests | $29,321·06 | $29,112·25 - $29,529·88 |
| ART adherence tests | $55,667·40 | $55,259·78 - $56,075·02 |
| Travel vouchers | $3,142·29 | $3,088·98 - $3,195·59 |
| Food vouchers | $1,467·19 | $1,447·61 - $1,468·77 |
| Total (All variables) | $239,835·95 | $238,760·81 - $240,911·08 |
| Total per participant (All variables) | $2,284·15 | $2,273·91 - $2,294·39 |
